# Mapping the Contact Sites of the *Escherichia coli* Division-Initiating Proteins FtsZ and ZapA by BAMG Cross-Linking and Site-Directed Mutagenesis

**DOI:** 10.3390/ijms19102928

**Published:** 2018-09-26

**Authors:** Winfried Roseboom, Madhvi G. Nazir, Nils Y. Meiresonne, Tamimount Mohammadi, Jolanda Verheul, Hansuk Buncherd, Alexandre M. J. J. Bonvin, Leo J. de Koning, Chris G. de Koster, Luitzen de Jong, Tanneke den Blaauwen

**Affiliations:** 1 Mass Spectrometry of Biomacromolecules, Swammerdam Institute for Life Sciences, Faculty of Science, University of Amsterdam, Science Park 904, 1098 XH Amsterdam, The Netherlands; w.roseboom@uva.nl (W.R.); hansuk.bu@psu.ac.th (H.B.); L.J.deKoning@uva.nl (L.J.d.K.); C.G.deKoster@uva.nl (C.G.d.K.); 2 Bacterial Cell Biology and Physiology, Swammerdam Institute for Life Sciences, Faculty of Science, University of Amsterdam, Science Park 904, 1098 XH Amsterdam, The Netherlands; madhvinazir@gmail.com (M.G.N.); N.Y.Meiresonne@uva.nl (N.Y.M.); tamimohammadi@gmail.com (T.M.); j.verheul@uva.nl (J.V.); 3 Computational Structural Biology, Faculty of Science-Chemistry, University of Utrecht, Padualaan 83584CH Utrecht, The Netherlands; a.m.j.j.bonvin@uu.nl; 4 Faculty of Medical Technology, Prince of Songkla University, Songkhla 90110, Thailand

**Keywords:** cell division, Z associated protein A (ZapA), Filamenting temperature sensitive Z (FtsZ), quadrupole time of flight mass spectrometer (QTOF), Fourier-Transform Ion Cyclotron Resonance mass spectrometry(FTICR), 1,4-*bis*(succimidyl)-3-azidomethylglutarate (BAMG)

## Abstract

Cell division in bacteria is initiated by the polymerization of FtsZ at midcell in a ring-like structure called the Z-ring. ZapA and other proteins assist Z-ring formation and ZapA binds ZapB, which senses the presence of the nucleoids. The FtsZ–ZapA binding interface was analyzed by chemical cross-linking mass spectrometry (CXMS) under in vitro FtsZ-polymerizing conditions in the presence of GTP. Amino acids residue K42 from ZapA was cross-linked to amino acid residues K51 and K66 from FtsZ, close to the interphase between FtsZ molecules in protofilaments. Five different cross-links confirmed the tetrameric structure of ZapA. A number of FtsZ cross-links suggests that its C-terminal domain of 55 residues, thought to be largely disordered, has a limited freedom to move in space. Site-directed mutagenesis of ZapA reveals an interaction site in the globular head of the protein close to K42. Using the information on the cross-links and the mutants that lost the ability to interact with FtsZ, a model of the FtsZ protofilament–ZapA tetramer complex was obtained by information-driven docking with the HADDOCK2.2 webserver.

## 1. Introduction

Cell division in Gram-negative bacteria requires the timed initiation of the invagination of the three-layered cell envelope and synthesis of two new cell poles [1]. Three stages can be discriminated during this process. First, the tubulin homologue FtsZ polymerizes into a Z-ring at midcell [2]. The second stage prepares the midcell position for cell pole synthesis and consists of recruitment of the division machinery and the redirection of peptidoglycan (PG) synthesis [3,4,5]. The last stage includes the synthesis of the new cell poles by the division machinery.

FtsA and ZipA anchor FtsZ polymers to the cytoplasmic membrane and assist the establishment of a Z-ring [6]. FtsZ was observed to move in a helical wave in the cytosol that transforms into the Z-ring at midcell. ZapA was shown to stimulate this transition [7] by, most likely, bridging of the protofilaments [8,9]. In addition, ZapA recruits ZapB to the Z-ring [10,11]. In the absence of ZapB, ectopic Z-rings and helices are formed, whereas overexpression of ZapB affects nucleoid morphology [12]. ZapB forms a connection via ZapA between FtsZ [10,13] and the MatP protein, which is bound to *matS* sites distributed close to the terminus region of the chromosomes [14,15]. Probably because of the binding of ZapB to MatP at midcell, ZapA is also able to localize to some extent at midcell without FtsZ [11]. The closure speed of the septum is enhanced in the absence of MatP, suggesting that its presence might assist in avoiding premature closure of the septum before the nucleoids are sufficiently segregated into the daughter cells [16]. Therefore, ZapA appears to have a double function: assistance in the initiation of cell division and recruitment of ZapB. Although the deletion of each protein individually causes only 21–25% Z-ring mislocalization in the population, deletion of both ZapA and ZapB causes more ectopic Z-ring formation (35%) [10].

The crystal structures of ZapA from *Pseudomonas aeruginosa* [17] and of *Escherichia coli* [18] are tetrameric, with each monomer consisting of a small globular amino terminal head followed by a long helix that forms a coiled-coil with a second ZapA monomer. The two dimer coiled-coils are intertwined, exposing the globular domains at the extremes of the dog-bone-like tetramer. The globular domain is thought to interact with FtsZ [18] and with ZapB [19].

FtsZ crystal structures are available from *Pseudomonas aeruginosa*, *Bacillus subtillis*, *Mycobacterium tuberculosis*, *Aquifex aeolicus*, and *Staphyloccocus aureus* [20,21], but these structures all lack the approximately 50–70 amino acids of the C-terminal variable region of the protein. This unstructured sequence ends in a conserved alpha-helical segment (C-terminal tail or CTT, in *E. coli* amino acids 369–379) followed by a short variable region (CTV, up to amino acid 383) that are important for many interactions between FtsZ and other cell division proteins [22,23,24]. For instance, ZipA as well as FtsA are reported to bind to the last 15 amino acids of FtsZ [25,26,27,28].

High concentrations (10–20 mM) of divalent cations cause the alignment of multiple FtsZ protofilaments in bundles, which is enhanced in the presence of a tetrameric ZapA [29]. When the concentration of divalent cations is kept lower (2–5 mM MgCl_2_), ZapA seems to be more involved in the bridging of FtsZ’s two protofilaments [8,9].The molecular details on where ZapA and FtsZ interact are not available and it is difficult to obtain them by co-crystallization as the protofilaments and bundles of FtsZ cannot be crystallized at present. Therefore, we have used chemical cross-linking in combination with mass spectrometry (CXMS) and site-directed mutagenesis to identify which amino acids of ZapA and FtsZ are engaged in their interaction.

## 2. Results

### 2.1. Optimization of Cross-Linking Conditions for Mass Spectrometric Analysis

**Figure 1:**
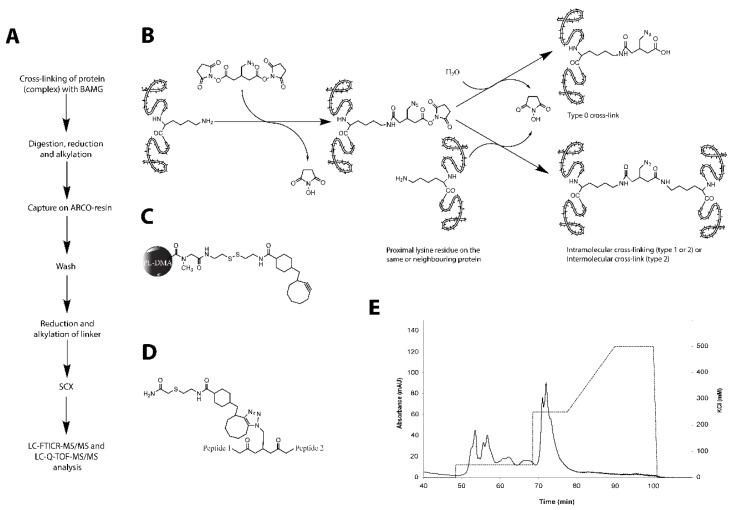
Overview of the used cross-linking methodology and terminology. (**A**) experimental work flow. Proteins are incubated with the azide-containing cross-linker *bis*(succinimidyl)-3-azidomethyl glutarate (BAMG). After cross-linking, proteins are digested and the obtained peptide mixture is incubated with the azide-reactive cyclooctyne (ARCO) resin to capture the cross-linked peptides out of the bulk of unmodified peptides. After cleavage from the resin, enriched peptides are fractionated by strong cation exchange (SCX) chromatography and analyzed by mass spectrometry. (**B**) structure of BAMG and its reactions with lysine residues in proteins. BAMG can react with a single lysine residue, the other reactive half of the cross-linker becoming either hydrolyzed or reacted with the quenching agent used to stop further cross-linking (type 0 cross-link), or it can react with two proximal lysine residues (type 1 or type 2 cross-linking). A type 1 cross-link occurs between two lysine residues in the same peptide after proteolytic digestion, while a type 2 cross-link connects lysine residues in different peptides. A type 2 cross-link can be formed in the same protein (intramolecular cross-linking) or between two different neighboring proteins (intermolecular cross-linking). BAMG adds 169.1 Da to a type 0 cross-linked peptide and 151.0 Da to type 1 and type 2 cross-linked peptides. (**C**) ARCO-resin, consisting of a poly-dimethylacrylamide solid support, a disulphide as a cleavable linker, and a cyclooctyne as a reactive group towards azides. Via the strain-promoted azide–alkyne cycloaddition, azide-containing peptides are captured on the resin. (**D**) enriched type 2 cross-linked peptides. The modification adds 509.2 Da to type 1 and type 2 cross-links and 527.2 Da to type 0 cross-links. (**E**) SCX chromatogram of enriched peptides. Type 0 and type 1 cross-linked peptides (solid line) elute predominantly at 50 mM KCl (dashed line), while elution of most type 2 cross-links occurs at a higher KCl concentration.

Electron microscopy images showed that the tetrameric ZapA protein is able to cross-link two single FtsZ protofilaments as well as two pairs of FtsZ protofilaments in vitro [8,29]. To determine which parts of the interacting proteins are involved in formation of this higher-order structure, an in vitro chemical cross-linking approach was chosen. As both proteins possess a number of surface-exposed lysines, amine cross-linking with a bifunctional *N*-hydroxysuccimidyl ester was used to determine the interaction domains. For this purpose, the cross-linker 1,4-*bis*(succimidyl)-3-azidomethylglutarate (BAMG) [30] was used, which enables isolation of cross-linked peptides by their coupling to azido-reactive cyclooctyne (ARCO)-modified polydimethyl acrylamide (PL-DMA) beads (Figure 1) [31].

The binding of GTP at the interface of two FtsZ monomers initiates polymerization provided that a critical concentration of FtsZ is present [32,33,34,35,36]. Polymerization was performed with 3.4 µM FtsZ and 6.8 µM ZapA at the physiological pH of 7.5 at 30 °C and in the presence of 5 mM MgCl_2_. The polymerization was detected by light scattering. After stabilization of the baseline, polymerization was initiated by the addition of 10 mM GTP in polymerization buffer to a final concentration of 60 μM. Because the maximum amount of light scattering was reached after 30 s (Figure S1A), this was the time point at which the cross-linker was added. At an FtsZ to ZapA ratio of 1:2, 100 μM discuccinimidyl glutarate (DSG) or BAMG (supporting information Figure S1B) resulted in the conversion of approximately 50% of both ZapA and FtsZ into products of which the molecular weight (MW) suggested cross-linking between ZapA molecules, between FtsZ molecules, and between ZapA and FtsZ. The BAMG cross-linked material was digested by trypsin and the cross-linked peptides were isolated using an azide-reactive cyclooctyne-conjugated resin (Figure 1) [31]. The peptides were released by reduction and alkylation of the linker, fractionated using ion-exchange chromatography, and subjected to LC-MS/MS for identification [31].

Three types of BAMG-conjugated peptides were identified, designated type 0, type 1, and type 2 [37]. In type 0 cross-linked peptides, also called monolinks, one lysine residue is modified by the cross-linker of which the second reactive ester group has been inactivated by hydrolysis, or by modification with Tris, used to stop the reaction. Two lysine residues cross-linked in the same peptide are designated type 1 cross-links, or looplinks. In type 2 cross-links or interpeptide cross-links, lysines from two different peptides are connected. Type 2 cross-links can be formed between peptides from different proteins or between peptides from the same protein remote from each other in the amino acid sequences of the protein. Type 2 cross-links are the most informative ones of the three from a three-dimensional (3D) structural point of view, since the linked amino acid residues must have been close together in space at the time of cross-linking depending on the length of the spacer of the used bifunctional reagent. The length of the spacer of BAMG is 7.7 Å. When fully stretched, the side chain of lysine has a length of 6.5 Å. Consequently, the maximal distance between C_α_ atoms of linked residues that can be spanned by BAMG is 20.5 Å.

In our cross-link experiments, we used conditions in which a mixture of ^14^N-FtsZ and ^15^N-FtsZ in a 1:1 ratio was polymerized. Under these conditions, a distinction can be made between intraprotein and interprotein cross-linked peptides from FtsZ. The pair of peptides in an intraprotein cross-link is either ^14^N- or ^15^N-labelled and therefore gives rise in a mass spectrum to two monoisotopic peaks of equal intensity with a mass difference that depends on the number of N atoms. In contrast, an interprotein cross-link between two peptides A and B is characterized by four mass peaks of equal intensity. One of the two additional peaks corresponds to a cross-link composed of ^14^N-labelled peptide A and ^15^N-labelled peptide B and the other one is composed of ^15^N-labelled peptide A and ^14^N-labelled peptide B. Three peaks are observed if A and B contain an equal number of N atoms. In the supporting information, Figure S1C shows that both isotopes of FtsZ polymerize equally well. To determine to which extent intermolecular FtsZ cross-links could be detected when FtsZ polymers form bundles in the presence of Ca^2+^, we choose conditions in which ZapA is absent in the reaction medium.

### 2.2. Cross-Links are Identified at a Low False Discovery Rate

Cross-linked peptides were identified as described in the Experimental procedures using a decoy database approach to determine the false discovery rate. This resulted in 16 different type 2 cross-links (Table 1) represented by 93 assigned spectra, while none of the decoy peptides fulfilled these criteria. The lack of decoy sequences indicates a low false discovery rate of identified cross-links. For annotated MS/MS spectra, see supporting information Table S1. The MS/MS spectra were subjected to a database search by Mascot against the *E. coli* database in order to identify unmodified peptides and type 0 and type 1 cross-linked peptides. More than 96% of the assignments above the Mascot threshold score for identity or extensive homology (*p* < 0.05) comprised FtsZ and ZapA peptides. This underscores the high purity of the proteins used in this study.

### 2.3. Cross-Links between FtsZ Molecules

**Figure 2:**
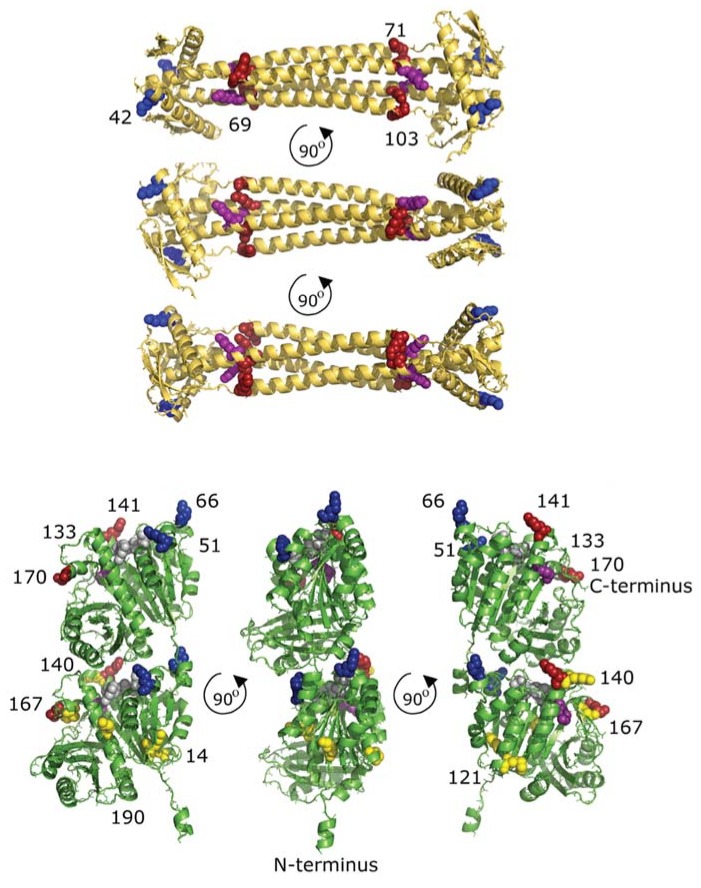
The ZapA structure (4P1M, [18]) in yellow and a model of the *Escherichia coli* FtsZ structure [38] in green with lysines involved in cross-links indicated. The K42 of ZapA cross-links with K51 and K66 of FtsZ (all blue). K71 and K103 (red) cross-link with each other and K71 cross-links with K69 (purple). In the upper molecule of the FtsZ dimer, the lysines that were found to cross-link are indicated. The C-terminal K380, which is not resolved in the model of the crystal structure, cross-links with K133 and K170 (both in red) and with K51 and K66. K141 in FtsZ also cross-links with K133 (purple). In the lower FtsZ molecule, the lysines that did not provide cross-links are indicated in yellow. The GTP connecting the two FtsZ monomers is shown in grey.

BAMG reacts with the majority of lysine residues under our experimental conditions. In FtsZ, no less than 14 out of the 16 lysine residues were detected in a form conjugated with BAMG. Seven out of 16 lysines of FtsZ were involved in formation of type 2 cross-linked peptides. Thirteen lysine residues were found to be involved in type 0 or 1 cross-linked peptides. For two lysine residues (K121 and K190), no evidence was obtained for conjugation with BAMG, but that does not necessarily mean that K121 and K190 are not surface-exposed. It could be that tryptic peptides with these residues involved in type 0, type 1, or type 2 cross-linking are too large or ionize insufficiently for detection by mass spectrometry. Alternatively, pK values of surface-exposed lysines may vary, implying that residues with a relatively high pK value of the side chain amine group are expected to react relatively poorly. Note that only the neutral -NH_2_ group can react with BAMG in a nucleophilic substitution reaction, while the protonated -NH^3+^ group is not reactive. (Figure 2).

For two cross-links, the distances between C_α_ atoms of interlinked residues could be determined in the model of the 3D structure of FtsZ (Figure 2). For the K141–K133 cross-link, this distance is less than the 20.5 Å that can be spanned by BAMG, while the K141–K66 cross-link exceeds this value by about 4 Å. Both are intraprotein cross-links, since mass spectra reveal that both their composing peptides are either labelled by ^14^N or ^15^N, while hybrid ^14^N–^15^N species are completely lacking (Figure S2). Therefore, the exceeding by 4 Å of the BAMG-spanning distance between the C_α_ atoms of the K141–K66 cross-link in the FtsZ model can be explained either by conformational flexibility in the peptide segments comprising the linked residues, or by possible coordination errors in the template structure, or by different conformations of template and target protein. Similarly, the cross-link between K42 of monomer 1 and K42 of monomer 2 in the globular head of ZapA exceeds the distance of the BAMG cross-linker. In solution, these two K42 residues apparently have the ability to be closer to each other than in the crystal structure.

**Figure 3:**
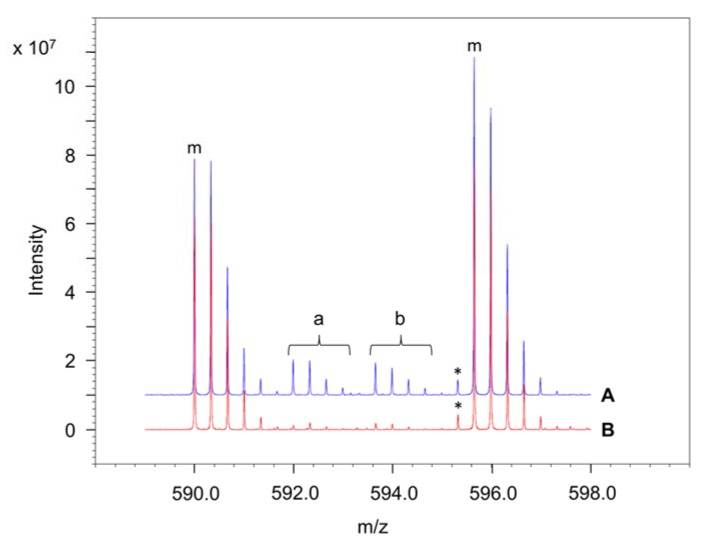
Fourier-transform ion cyclotron resonance (FTICR) mass spectra of the cross-link formed in FtsZ between K380 and K170. In the presence of ZapA, only intraprotein cross-links are formed (trace B). In the absence of ZapA and in the presence of Ca^2+^, a mixture of intraprotein and interprotein cross-links are formed (trace A). Cross-link experiments were carried out with ^14^N- and ^15^N-labelled FtsZ added to the reaction medium in a 1:1 ratio. The monoisotopic peaks in the ^14^N- (left) and ^15^N-(right) labelled peptides are marked m. Composing peptides of an intramolecular cross-link are either both ^14^N-labelled (A, left spectrum) or ^15^N-labelled (A, right spectrum). The peak marked with the asterisk indicates that the extent of ^15^N labelling was about 95%. The presence of interprotein cross-links is revealed by the hybrid ^14^N(LLKVGLR)–^15^N(KQAD) (a) and the hybrid ^15^N(LLKVGLR)–^14^N(KQAD) (b) spectra. A pure interprotein cross-link is revealed by a 1:1:1:1 peak intensity ratio of the four spectra. In a mixture of intraprotein and interprotein cross-links, the hybrid spectra have a lower intensity than the ^14^N and the ^15^N spectra.

One of the composing peptides in the other seven FtsZ cross-links comprises either the C-terminal tryptic peptide KQAD (residue 380–383) or the peptide with the nearby residue K367 or both these peptides. Both cross-linked residues in these peptides, i.e., K380 and K367, are in the C-terminal stretch of about 55 amino acids, of which the structure is not resolved in FtsZ crystals, and, therefore, is thought to be largely disordered [39,40]. In the presence of ZapA, all cross-links in which these C-terminal residues participate are intraprotein species, since ^14^N–^15^N hybrid cross-peptides are largely lacking (Figure 3 and Figure S2C–J). Apparently, the extension of the disordered C-terminal tail is not enough under these conditions to enable the residues K380 and K367 to form intermolecular covalent linkages in the presence of BAMG with a neighboring FtsZ molecule.

We did not identify interprotein FtsZ cross-links formed in the presence of ZapA (Table 1), whereas SDS-PAGE analysis shows the formation of covalently linked FtsZ dimers, trimers, and possible higher-order FtsZ polymers under these conditions (Figure S1B, right panel, lane 4). Interestingly, in the presence of Ca^2+^, interprotein FtsZ cross-links were observed (Figure S2), indicating that the bundling of FtsZ induced by Ca^2+^ resulted in a different higher-order structure than that induced by ZapA. This is also illustrated by the observation that the interprotein cross-links found in the Ca^2+^-containing sample were found as intraprotein cross-links in the sample containing ZapA but not Ca^2+^. This can be explained by assuming that under bundling conditions FtsZ protofilaments align close enough to enable C-terminal lysines to form intermolecular cross-links between two opposing FtsZ strands, whereas the presence of ZapA prevents their association by bridging the FtsZ filaments.

### 2.4. Interprotein Cross-Links of ZapA

In ZapA, all four lysine residues were involved in type 2 cross-linking resulting in five identified cross-linked peptides (Figure 2). Three of these must be interprotein cross-links since the cross-links comprise either two identical linked lysine residues, namely K42-K42 and K103-K103, or two peptides with overlapping sequences, i.e., the cross-link between K69 and K71 (Table 1). These three intermolecular cross-links fit in a ZapA dimer with two parallel coiled-coil regions. It is noteworthy that the distance between both K42 residues in the model based on the crystal structure of ZapA [18] is 6.4 Å more than the maximum possible distance between BAMG cross-linked lysines, suggesting flexibility in the head domain of a ZapA dimer. The possible distances in the other two cross-links, comprising residues K42 and K103 and residues K70 and K103, are much too large in the crystal structure of ZapA for cross-linking in a dimer with two parallel coiled-coil regions. However, the cross-links fit well by assuming a linkage between two anti-parallel arranged ZapA dimers. These results indicate that ZapA forms tetramers, in agreement with previously obtained evidence about the quaternary structure of ZapA under similar FtsZ polymerization conditions [8].

### 2.5. Cross-Links between ZapA and FtsZ

**Figure 4:**
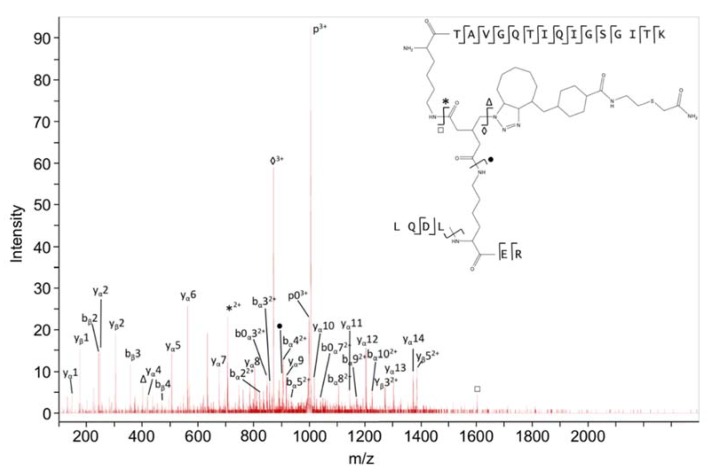
Q-TOF MS/MS spectrum of a cross-linked peptide with K42 from ZapA connected to K51 from FtsZ. Product ions from peptide α and peptide β are annotated according to [37]. The inset shows the structure of the cross-linked peptide, the positions of cleaved bonds, and types of produced ions. P, precursor ion; p0, precursor ion with H_2_O loss. Five products resulting from cleavages in the cross-link remnant are indicated in the structure and corresponding mass peaks by square, star, triangle, diamond and black dot.

Two cross-links were found between ZapA and FtsZ (ZapAK42-FtsZK51 (Figure 4) and ZapAK42-FtsZK66). K42 of ZapA is located in the globular head domain of this protein, while the residues K51 and K66 of FtsZ are close enough in space in an FtsZ monomer to explain cross-linking by one defined binding site between ZapA and FtsZ. Alternatively, each K42 of ZapA was cross-linked to a different FtsZ monomer. It is well-known that FtsZ molecules form protofilaments in a head-to-tail arrangement of monomers [41]. Residues K51 and K66 are close to the interface between two monomers and are also in the vicinity of the GTP binding site. Interestingly, evidence has been presented that ZapA not only cross-links FtsZ protofilaments but also stimulates longitudinal FtsZ–FtsZ interactions [9]. This can be explained by assuming binding of ZapA to the FtsZ–FtsZ interface, thereby stabilizing the FtsZ–FtsZ interaction. ZapA has been reported to reduce the rate of GTP hydrolysis by FtsZ under conditions that stimulate bundling (10 mM Mg^2+^ or 10 mM Ca^2+^) [42], but not under non-bundling conditions (5 mM MgCl^2+^) [8]. Therefore, a possible explanation could also be that binding of ZapA close to the GTP binding site may modulate the GTPase activity, thereby regulating protofilament formation.

### 2.6. ZapA Mutants

To obtain more detailed information on the interaction between FtsZ and ZapA and to be able to verify the in vitro information, several ZapA mutants were made of residues in the vicinity of the cross-linked K42 (Table 2).

**Figure 5:**
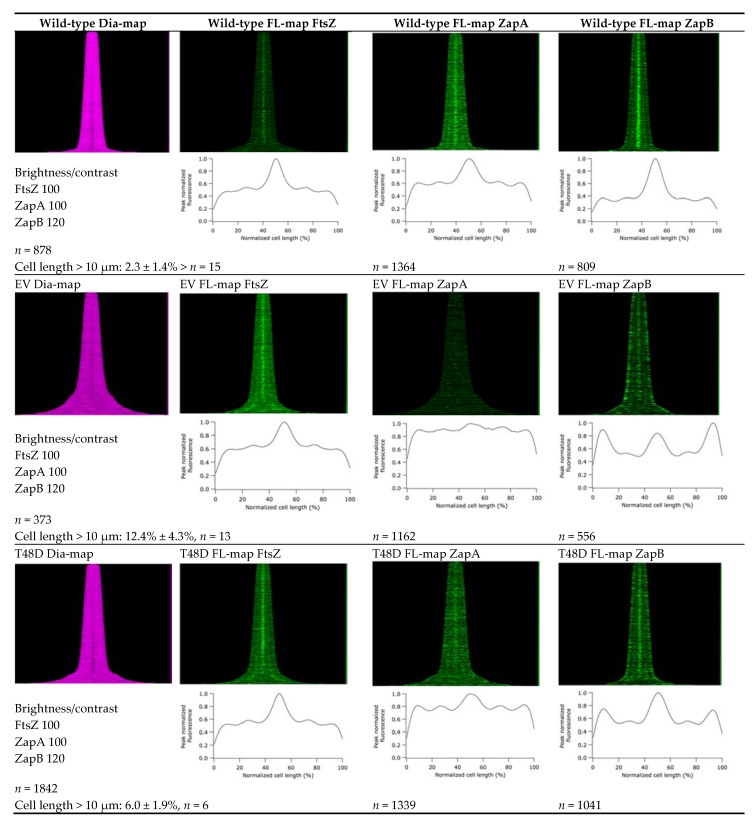
Map of diameter and map of fluorescence profiles and average fluorescence profile of FtsZ, ZapA, and ZapB of TB28∆zapA [10] expressing ZapA mutants from plasmid. The cells were grown exponentially in rich medium at 37 °C and the OD600 was kept below 0.3. Expression of the mutants was induced with 50 µM IPTG for six mass doublings. Cells were fixed and immunolabeled as described in the experimental procedures. Of each sample, from left to right the map of diameter of the FtsZ-labeled sample, the map of fluorescence of the FtsZ-labeled sample, the map of fluorescence of the ZapA-labeled sample, and the map of fluorescence of the ZapB labeled sample is shown. The cells are sorted according to their length in the maps (small cells on top and the longest cell on the bottom of the map). The number of analyzed cells is indicated and the percentage of cells with a cell length of more than 10 µm and the S.E.M. from a number (*n*) of experiments as indicated are shown. Brightness and contrast reflect the grey values within the map of fluorescence. EV is TB28∆zapA containing the plasmid pTHV037 [4] with the same resistance marker but without the *ZapA* gene. All mutants are shown in the supporting information Figure S4.

**Figure 6:**
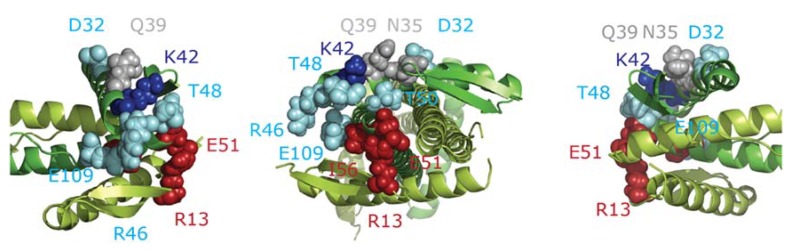
Weakly and non-complementing mutants in ZapA. The *E. coli* ZapA tetramer [18] is shown with two molecules in limon and two in green. The weakly complementing mutant amino acid residues (D32, T48, R46, E109) made in the present study are shown in cyan, the non-complementing residues (R13, E51, I56) are in red, and the complementing residues (N35 and Q39) in grey. Residue K42, which was cross-linked to FtsZ and which mutated versions were weakly complementing, is shown in blue.

After establishing the conditions for complete complementation by wild-type (WT) ZapA expressed from plasmid in strain TB28ΔzapA (see Materials and Methods, Figure S3), the ZapA mutants were expressed in exponentially growing cells in Trypton Yeast (TY) at 37 °C at the suboptimal IPTG concentration of 50 μM to make sure that we would not overlook mutants with a reduced affinity due to overproduction. Mutations in ZapA could also affect the interaction with ZapB. Therefore, the cells were fixed and immunolabelled with antibodies against ZapA, FtsZ, and ZapB. In the absence of ZapA, FtsZ localizes less pronounced at midcell with more cytosolic FtsZ background than in a wild-type cell. In the absence of ZapA, ZapB localizes at the poles and near the newly synthesized septa whereas it localizes very focused at midcell in the presence of ZapA (Figure S4). Mutants D32A, N35A, N35D, Q39A, Q39E, Q39K, and T48R complemented the ΔzapA strain, localized at midcell, and were able to recruit ZapB (Table 2, Figure 5, supporting information Figure S4). Mutants D32K, K42A, K42E, R46E, T48D, T50RE51D, and E109K mildly affected the length of the cells with between 2.5% and 6% of the cells having a cell length of more than 10 µm (Table 2, Figure 5, Figure S4). In these cells, the balance between midcell localization of ZapA and ZapB and the cytosolic presence of these proteins shifts towards ZapB. The percentage of cell with a cell length of more than 10 µm of the ZapA mutants R13D, E51K, and I56K was between 6% and the same value as that of cells expressing an empty vector, indicating that they did not complement the ΔzapA strain (Table 2, Figure 5, Figure S4), which is also confirmed by the mislocalization of ZapB in the old and new poles (Figure 5, Figure S4). Residues R13, D32, R46, T48, E51, and I56 are grouped in or close to a groove in the vicinity of the K42 cross-linking site (Figure 6), whereas E109 is at the bottom of the groove. Therefore, we assume that this area is important for the FtsZ binding site or for the correct folding of this site.

### 2.7. Docking of FtsZ and ZapA

**Figure 7:**
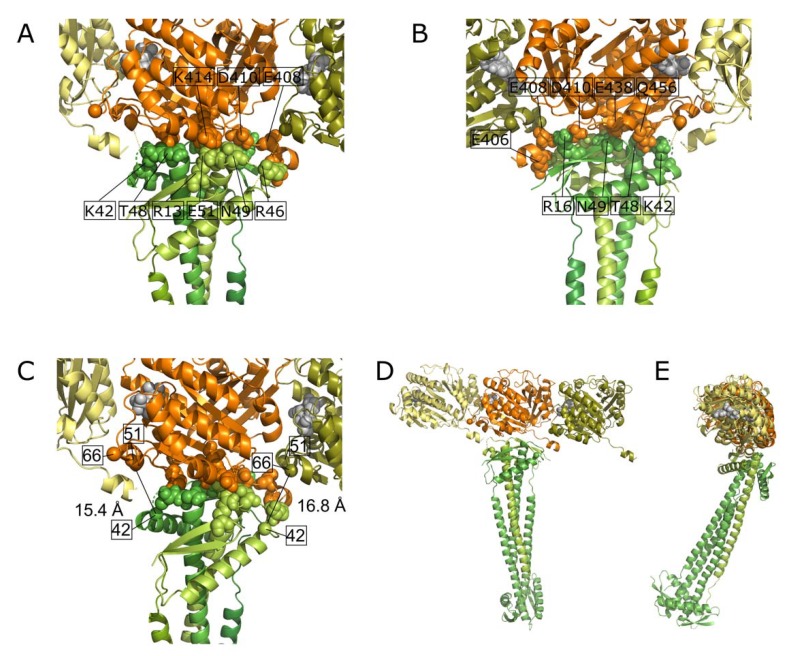
Haddock model of the interaction between a FtsZ filament and the ZapA tetramer. (**A**) and (**B**). Front and back side view of a closeup of the interaction site with the interacting amino acid residues of both proteins indicated. (**C**) Distances between the C_α_ atoms (spheres) of residue K42 of ZapA and residues K51 and K66 of FtsZ that were found to cross-link. (**D**) Front and side view of the interacting proteins. (**E**) Frontal overview of a FtsZ filament consisting of three monomers and the interacting ZapA tetramer. (**D**) Side view of the same molecules with a second FtsZ filament and the cytoplasmic membrane indicated. The grey spheres at the interface of the FtsZ monomers is GTP.

A model of the complex was obtained by information-driven docking with the HADDOCK2.2 webserver [43] (see Methods). For this, a trimer of *E. coli* FtsZ based on homology structure prediction [38] was docked against the *E. coli* ZapA tetramer (4P1M, [18]) using the mutation data and the two intermolecular cross-links as ambiguous distance restraints (See Table S2). [44]. Because of the presence of multiple K42 in the ZapA tetramer and also multiple possible K51 and K66 in the trimer repeat model of FtsZ, the cross-link restraints were defined as ambiguous distance restraints between those, effectively allowing HADDOCK to select the best pair combination. From the identified putatively important residues for binding from the mutagenesis study, we used R13, R46, T48, and E51 to drive the docking. Ambiguous interaction restraints were defined from those together with their symmetry-related residue in the same interface in another ZapA monomer to all solvent-accessible residues of the central FtsZ monomer in the trimer repeat. E9 and I56 were excluded because they are poorly solvent-accessible and E109 was excluded because of its remote position from the patch formed by the other residues. The docking resulted in 17 clusters (see statistics in Table S3). The top cluster (#5) contains the overall best scoring model (HADDOCK score of −170 (a.u.). It satisfies the two cross-links with C_α_–C_α_ distances of ~16 Å and involved two consecutive FtsZ monomers. The list of interacting amino acid residues for the best model is given in Table S4. The FtsZ filament binds to the front of the globular domain of ZapA. Both proteins are almost in the same plane at an angle of about 70° (Figure 7). Contact sides of ZapA are the loop 46–51 of one monomer and the residues R13 and R16 in the other monomer that form together the globular domain of the dog-bone-shaped protein. The majority of the interacting residues of FtsZ are from one monomer (residues E406, T408, D410, K414, E438, G455, Q456, A490, E493, and G494 (Table S4)).

## 3. Discussion

Using the BAMG cross-linker, which allows for the MS/MS sequencing of the individual cross-linked peptides, the interaction sites between FtsZ and ZapA were studied in vitro under non-FtsZ protofilament bundling conditions. The with high significance detected Type 2 cross-links that are formed between either two lysines of different protein molecules (intermolecular) or between two residues within one protein (intramolecular) corroborate a number of observations. In the presence of ZapA, no intermolecular cross-links between FtsZ molecules were found. A number of intermolecular cross-links were found in the absence of ZapA but in the presence of Ca^2+^ that stimulates bundling of FtsZ. This suggests that the initial activity of ZapA is to bridge FtsZ without aligning the protofilaments into bundles. This is in agreement with super resolution images of the FtsZ ring in bacteria [16,45,46]. In these studies, the ring is depicted as consisting of short protofilaments of up to 100 nm that are randomly oriented in a structure that has a thickness of about 60 nm and a width of about 100 nm. For ZapA molecules, the thickness and width of the ring structure was determined to be 85 and 45 nm, respectively [16]. The thickness and width of both the Z-ring and the ZapA “ring” do not change [16] but the density of the FtsZ molecules in the ring increases during constriction [16,47]. Depending on whether ZapA density changes in parallel, the number of FtsZ filaments that are bound by ZapA might change during septum closure.

The C-terminal 57 amino acids sequence of FtsZ that is not resolved and often not even present in FtsZ crystal structures was found to form only intramolecular cross-links with residues present in a restricted area with a diameter of only 38.6 Å on one side of the protein in the presence of ZapA. The confinement of K380 to only intramolecular cross-linking suggests that residues in the flexible tail find themselves in a relatively small volume most of the time as suggested by [40]. Because ZapA binds to the other side of FtsZ, it seems unlikely that ZapA would be responsible for the confinement of the flexible tail. Possibly, the tail adopts multiple conformations in a limited space that disrupt crystal formation. The amino acid sequence 326–369 in the disordered C-terminal tail of FtsZ is not important for its function. However, a length between 43 and 95 amino acids is a requirement [40]. This suggests that, in the cell, the binding of FtsA or ZipA to the conserved 11 amino acids at the end of this tail could increase the freedom of the whole fragment and that this flexibility in the length of the spacer facilitates the constriction process. The formation of intermolecular cross-links involving K380 under bundling conditions in the absence of ZapA and in the presence of Ca^2+^ may occur between neighboring FtsZ protofilaments.

Thus far, it was not known what the interface of the FtsZ–ZapA interactions is. Our results provide a first indication as FtsZ K51 as well as FtsZ K66 were found to cross-link to K42 of ZapA. Using site-directed mutagenesis, we have identified a number of residues in the vicinity of K42 in the globular head of ZapA that included the weakly and non-complementing mutant residues R13D, D32A/K, R46E, T48D, E51K, I56K, and E109 (Figure 6 and Figure S4). This area seems to be the most likely candidate for the binding site of FtsZ. On the frontal side of the globular ZapA domain, a long alpha helix can be found that was also reported [18] to be the interaction site for FtsZ based on site-directed mutagenesis. However, we have mutated D32 to A and K, one of the residues that was mutated (D32A) in the Roach et al. study, and found that the mutant’s morphology was only weakly disturbed with just 3% of the cells having a length of more than 10 μm and were still able to bind FtsZ to a large extent (supporting information Figure S4). Also, mutation of residues N35 and Q39 in this helix had no effect on the interaction between ZapA and FtsZ. Based on our results, we therefore must conclude that amino acids N35 up to Q39 of ZapA are not involved in the binding site of FtsZ. An explanation for the contradicting results by [18] and our mutagenesis study that we can envision is that we use untagged ZapA whereas in the other report a His-tagged ZapA was used. In our experience, the His-tagged version is not fully functional [8]. However, the two His-tags differ in that the His-tag of [18] has a factor XA proteolytic site and was shown when fused to a wild-type ZapA to be able to complement a ∆*zapA* strain, whereas the His-tagged ZapA of [8] had an enterokinase proteolytic site and was not able to complement such a strain. Possibly, the wild-type ZapA with a functional His-factor-XA-tag could in the case of the mutants enhance the effect of the mutations due to steric hindrance. Alternatively, the His-tagged version of ZapA might reveal weak binding sites due to its destabilizing effect. In the His-tagged version of ZapA case, residues 28–33 of the helix might be part of a binding site for FtsZ. Sedimentation and electron microscopy studies of [18] using isolated His_6_-ZapA mutants showed that the mutants in the C-terminal part of the helix (residues 28, 32, 33, and 46) had a 70% reduction in affinity for FtsZ and also a reduction of 20% in FtsZ bundling capacity in vitro. However, using the information on the observed cross-links and mutants and the HADDOCK software for protein–protein interactions, the obtained model does not find an interaction between the C-terminal helix of ZapA and FtsZ. Another ZapA mutant N60Y was reported to have lost the ability to interact with FtsZ [11]. This residue can be found between the coiled-coil domain and the globular head of the ZapA dimer adjacent to the C-termini of the second ZapA dimer that form together a tetramer. Based on the HADDOCK model (Figure 7), it does not seem to be part of the ZapA–FtsZ interface unless a second binding site exists. Given the delicate position of this residue, the N to Y mutation might destabilize the tetramer and shift the equilibrium more to the formation of dimers, such as the I83E mutants described by [29].

In this model (Figure 7), the top of the globular domain of ZapA binds to the FtsZ filament with the majority of the interactions with one of the FtsZ monomers. Residue R46A of ZapA [18] was not complementing and R46E in our study was not fully functional suggesting that it is part of the FtsZ binding site, which is indeed observed in the model. Other residues that were mutated and showed in vivo localization defects were T48D, E51K, and R13D that were found to interact with FtsZ in the model. I56K and E109 seem not to be involved in FtsZ binding. Possibly, these mutations affected the folding of the binding site, reducing the affinity for FtsZ. Minimal medium-grown *E. coli* cells have about 1100 ± 77 FtsZ molecules at midcell that are organized as ~45 protofilaments of variable length up to 100 nm and 40 ± 3 ZapA tetramers at midcell [16,47]. Sufficient ZapA tetramers are available to bind and cross-link all FtsZ protofilaments into a three-dimensional network. In such an arrangement, cross-linking of two FtsZ protofilaments by a ZapA tetramer seems to be realistic. In a recent super resolution study, mEos2-ZapA clusters were found to occupy a slightly smaller zone than FtsZ-mEos2 [16], suggesting that ZapA is surrounded by FtsZ protofilaments during cell constriction, which would support a model in which FtsZ filaments could also be bound by one ZapA tetramer.

## 4. Materials and Methods

### 4.1. Bacterial Strains and Growth Conditions

FtsZ was isolated from *E. coli* strain BL21(DE3) pRRE6 [48]. DH5α [49] was used to isolate plasmid DNA. Strains TB28 and TB28Δ*zapA* [10] were used to determine whether ZapA mutants were able to complement the absence of wild-type ZapA. Cells were grown in Trypton Yeast broth (TY) at 37 °C or in glucose minimal medium (GB1) at 28 °C. For ^15^N-isotope labeling, Ca(NO_3_)_2_.4H_2_O was replaced by CaCl_2_ and (^15^NH_4_)_2_SO_4_ was used to grow the cells in GB1 medium for ^15^N-FtsZ isolation (see supporting experimental procedure for details).

### 4.2.Site-Directed Mutagenesis and Plasmid Construction

Plasmid pGP021 [8] was used as template for site-directed mutagenesis using the Quick change mutagenesis method (Stratagene, la Jolla, CA, USA) using the primers listed in supporting information Table S5. The plasmid expresses ZapA from a weakened *trc* promotor, which is IPTG inducible. Wild-type ZapA was expressed from plasmid in TB28Δ*zapA* using a range of IPTG concentrations (0, 10, 20, 50, and 100 µM). Based on the average length of the cells in the culture, induction with 50 μM IPTG for six mass doublings gave complete complementation. All subsequent experiments were induced using 50 μM IPTG for six mass doublings after which the culture was fixed by formaldehyde (final concentration 2.8%, Sigma-Aldrich, St. Louis, MS, USA) and glutaraldehyde (final concentration 0.4%, Merck, Kenilworth, NJ, USA) and immunolabelled as described [50] with antibodies against ZapA [8], FtsZ [51], and ZapB [10]. The ZapA antiserum was routinely purified by immunolabelling of TB28Δ*zapA* cells to adsorb potential cross-reactive IgG. The supernatant was subsequently used to label the ZapA mutants.

### 4.3. Microscopy and Image Analysis

For immunolocalization imaging, the cells were immobilized on 1% agarose [51] and photographed with a CoolSnap *fx* (Photometrics) charge-coupled device (CCD) camera mounted on an Olympus BX-60 fluorescence microscope through an UPLANFl 100×/1.3 oil objective (Olympus, Tokyo, Japan). Images were taken using modified acquisition software that used the program ImageJ by Wayne Rasband and analyzed using Object-J’s Coli-Inspector [47] (see for details supporting experimental procedures).

### 4.4. Cross-Linking

FtsZ was isolated as described [52] and ZapA was isolated using a His-tagged version after which the His-tag was removed by proteolysis as described [8]. Polymerization of FtsZ was studied by light scattering in 1200 μL polymerization buffer (50 mM HEPES, pH 7.5, 50 mM KCl, 5 mM MgCl_2_), 3.4 µM FtsZ, and 6.8 µM ZapA at 30 °C. After stabilization of the baseline, polymerization was initiated by the addition of 60 μM GTP (final concentration) in polymerization buffer. Alternatively, ZapA and GTP were mixed first and the reaction was initiated by the addition of FtsZ. For the experiments under bundling conditions, ZapA was omitted and the polymerization reaction contained 10 mM CaCl_2_. Polymerization in these experiments was initiated by the addition of 30 μM GTP. Cross-linking experiments were conducted using the same concentrations of FtsZ, ZapA, and GTP as in the light scattering assays. Shortly before addition to the polymerization reaction, 0.1 mg of 1,4-*bis*(succimidyl)-3-azidomethylglutaraat (BAMG, [30]) was dissolved in 13 µL acetonitrile (ACN). After 30–60 s of polymerization reaction (depending on the sample), 100–150 μM BAMG was added and the sample was briefly vortexed. Discuccinimidyl glutarate (DSG) instead of BAMG was used as a cross-linker for samples to be subjected to SDS-PAGE analysis. DSG has the same cross-linking efficiency as BAMG [30]. The sample was left to incubate for 30 min at 30 °C after which the cross-linking reaction was stopped with 10 mM Tris-HCl, pH 7.5.

The sample was concentrated on a 5-kDa cut-off BioMax filter to a volume of 50 μL. To alkylate cysteines, 100 μL freshly prepared denaturation solution (9 M urea, 30 mM iodoacteamide, 50 mM Tris-HCl, pH 8) was added and the preparation was incubated at room temperature (RT) for 30 min. The sample was subsequently diluted with 50 mM Tris-HCl (pH 8), reducing the urea concentration to 2 M, and digested with trypsin (Trypsin Gold, Promega, Madison, WI, USA) ((*w*/*w*) 1:20) for 3 h at 37 °C. The proteolysis was stopped by the addition of 50 μL 10% TFA, and the sample was stored in liquid nitrogen.

### 4.5. Isolation of Cross-Linked Peptides

The isolation procedure for cross-linked peptides was used as described previously [31] with minor modifications (see supporting experimental procedures).

### 4.6. Determination of the Amounts of Interprotein and Intraprotein Cross-Links Using ^14^N- and ^15^N-Labelled Peptides

In our approach to determine for each cross-link comprising FtsZ peptides the ratio of interprotein and intraprotein linkages, we mixed equal amounts of ^14^N- and ^15^N-labelled proteins in the cross-linking medium [53]. In intraprotein links, both composing peptides A and B are either ^14^N- or ^15^N-labelled, giving rise to a mass spectrum containing two isotope envelopes with a mass difference between the monoisotopic ^14^N and ^15^N peaks depending on the number of N atoms in the cross-linked peptide pair. An interprotein cross-link between two peptides A and B is characterized by four isotope envelopes of equal intensity, one of the two additional peaks corresponding to a cross-link composed of ^14^N-labelled peptide A and ^15^N-labelled peptide B and the other one composed of ^15^N-labelled peptide A and ^14^N-labelled peptide B. Three peaks are observed if A and B contain an equal number of N atoms. For each cross-link, the ratio of interprotein and intraprotein species was assessed by determining the best fit with a measured spectrum. Isotope patterns were calculated with EnviPat Web 1.6 (available online: http://www.envipat.eawag.ch/index.php) from the chemical formula, which can contain a mix of isotopes (for example ^14^N in one peptide and ^15^N in the other one). For calculations, we assumed a mass resolution M/∆M = 35,000, in which ∆M is the peak width at half maximal height, and 100% efficiency of ^15^N incorporation, which is close to the actual 95% efficiency. From the cross-linked peptide candidates, the chemical formula (Hill-sorted sum formula) was determined with a Bruker Compass Isotope Pattern combined with the chemical formula for the BAMG-cyclooctyne moiety (C_26_H_31_N_5_O_4_S) in four combinations ^14^N-peptide A + ^14^N-peptide B, ^14^N-peptide A + ^15^N-peptide B, ^15^N-peptide A + ^14^N-peptide B, and ^15^N-peptide A + ^15^N-peptide B.

Fitting of the calculated isotope patterns was determined by visual inspection against the measured data. The four generated isotope patterns were saved as a comma-separated value list and combined in Excel with the measured data. A correction for the intensity was made to fit the theoretical isotope data with the measured data. Relative intensities of the four combinations were calculated from the sum of the corrected theoretical isotope patterns.

### 4.7. Identification of Type 2 Cross-Linked Peptides

For identification of isolated cross-linked peptides, we used a previously described method by [54]. In this approach, the search engine Mascot is used for screening LC-MS/MS data against a database of all possible combinations of cross-linked peptide pairs in the form of linearized peptide pairs variably modified at lysine residues with the mass of the cross-linker remnant.

For identification of type 2 cross-links (Figure 1), a database of all possible cross-linked peptides for interrogation by Mascot was generated by xComb version 1.2 [55]. To this end, both the forward and reversed amino acid sequences of FtsZ and ZapA of interest were uploaded in UniProt FASTA format and trypsin was the chosen enzyme for digestion with two missed cleavages allowed. Both intra and interprotein cross-links were taken into account. The minimum peptide length for each peptide of the pair was two amino acids with at least one trypsin missed cleavage for amine cross-linking. The cross-link database was uploaded in Mascot version 2.2. The following parameters were used to identify candidate type 2 cross-links based on the processed data files from LC-MS/MS experiments: (i) a “nocleave” enzyme with the nonexisting amino acid “J” as the cleavage site; (ii) one missed cleavage; (iii) a fixed modification in the form of carbamidomethyl at C; (iv) a variable modification at K with a group composition of C_26_H_33_N_5_O_5_S; and (v) oxidized methionine as a variable modification. With FTICR mass spectrometry, the precursor mass tolerance was set at 20 ppm and the product ion mass tolerance at 0.05 Da. When searching with MS/MS data obtained with a Quadruple time of flight (Q-TOF) mass spectrometer, the mass tolerance of both precursor and products ions was set at 0.4 Da. Identified unmodified peptides and type 0 cross-linked peptides were used for internal mass calibration. No threshold score was taken into account for nomination by Mascot of candidate type 2 cross-linked peptides. For validation of type 2 cross-linked peptides nominated by Mascot, we developed the software tool Yeun Yan [56]. For proposed candidate cross-linked peptides, Yeun Yan calculates the masses of possible b and y fragments, b and y fragments resulting from water loss (b0, y0) and ammonia loss (b*, y*), fragment ions resulting from cleavage of the amide bonds formed in the cross-link reaction, and b, b0, b*, y, y0, and y* fragments resulting from secondary fragmentations of products of cross-link amide bond cleavages. An ions score (Yeun Yan score) is calculated according to the equation:

YY score = f_assigned_/f_total_ × 100%, in which f_assigned_ is the total number of matching fragment ions at 50 ppm (FTICR) or 200 ppm (Q-TOF) mass accuracy, and f_total_ is the total number of fragment ions in the spectrum taken into account, starting from the fragment ion of highest intensity, with a minimum of 10 fragments. Criteria used for assignment of type 2 cross-links were (i) a mass tolerance window of 7 ppm (FTICR) or 200 ppm (Q-TOF) for the intact peptide ion, (ii) detection of at least five unambiguous fragment ions at 0.05 Da (FTICR) or 200 ppm (Q-TOF) mass accuracy for cross-linked peptides, (iii) detection of at least two fragments at 50 ppm (FTICR) or 200 ppm (Q-TOF) mass accuracy for each composing peptide, and (iv) a YY score of at least 35. No fragment ions from a composing peptide with only two amino acids are required for a candidate type 2 cross-link if all other criteria for assignment are fulfilled and if the expected number of N atoms is in agreement with the mass difference between the ^14^N- and ^15^N-labelled candidate. If more than one candidate for a particular precursor ion is put forward, only the candidate with the highest score is assigned. If both candidates have equal scores, none of them will be assigned.

### 4.8. Determination of the False Discovery Rate (FDR)

The following definition of FDR is used: FDR = (FP/TP + FP) × 100%, in which FP (false positives) is the number of assigned decoy peptide MS/MS spectra and TP (target peptides) is the number of assigned target peptide MS/MS spectra. In decoy peptides, one or both composing peptides have a reversed sequence, while in target peptides both composing peptides have forward sequences.

### 4.9. Docking

The HADDOCK2.2 web server [43,44] was used to generate a model of the interaction of FtsZ with ZapA. All molecular images were produced using PyMOL. Docking of FtsZ and ZapA was performed by the HADDOCK software. The two intermolecular cross-links were defined as ambiguous distance restraints between the two symmetry-related LYS42 of ZapA and the three LYS51 and LYS66 of the three FtsZ monomers in the trimeric model used as the starting point for the docking. A trimer model of FtsZ was used for docking to mask the FtsZ–FtsZ interfaces in the fiber. Ambiguous interaction restraints were defined from the mutated residues on ZapA (R13, R46, T48, E51) (considering both possible residues at the interface because of the internal symmetry) to the solvent-accessible residues of the central FtsZ monomer in the trimer repeat model using a 40% relative solvent accessibility as calculated using NACCESS [57] (see Table S2). Since ZapA is a tetramer, for the docking, since HADDOCK only accepts a single chain per model, the number of the 2nd, 3rd, and 4th monomers was shifted by 200, 400, and 600, respectively. Similarly, a residue number shift of 400 and 800 was applied to the 2nd and 3rd FtsZ monomers, respectively. Default HADDOCK settings were used for the docking, generating 1000/200/200 models from the three subsequent stages of HADDOCK, namely rigid-body docking, semi-flexible refinement, and final refinement in water. The final models were clustered based on the fraction of common contacts [57] using a 0.75 cutoff. The HADDOCK parameter web-file S1 containing all input data and settings is provided as supplementary material together with the PDB coordinates of the best model (PBP S1 cluster 5.1).

## 5. Conclusions

- BAMG X-linking yields inter and intracross-links very accurately.
- ZapA keeps FtsZ protofilaments apart as no X-links between two different FtsZ molecules were found in the presence of ZapA. In contrast, under protofilament bundling conditions, i.e., in the presence of Ca^2+^, a number of cross-links between different FtsZ molecules was found.
- The structurally disordered C-terminal 55 amino acids of FtsZ occupied a limited space and are likely not extended in the absence of other cell division proteins.
- Cross-links confirm the tetrameric structure of ZapA in solution.
- The FtsZ filament binds to the front of the globular domain of ZapA. Both proteins are almost in the same plane at an angle of about 70°. Sufficient ZapA is present in the cells to cross-link most FtsZ protofilaments.

## Figures and Tables

**Table 1:** Overview of identified cross-linked peptides.

Protein(s)	Linked Residues	A Peptide	B Peptide	(Å)	ZapA, No Ca^2+^		No ZapA, Ca^2+^	
					Spectral Counts ^b^	Type	Spectral Counts	Type
FtsZ	141–66	**K**R	TAVGQTIQIGSGIT**K**GLGAGANPEVGR	24.8	11	Intra	6	Intra
FtsZ	141–133	**K**R	DLGILTVAVVT**K**PFNFEGK	12.3	2	Intra	–	–
FtsZ-FtsZ	141–367	**K**R	VVNDNAPQTA**K**EPDYLDIPAFLR	u	3	Intra	4	Intra
FtsZ-FtsZ	141–380	**K**R	**K**QAD	u	3	Intra	9	Mix
FtsZ-FtsZ	170–367	LL**K**VLGR	VVNDNAPQTA**K**EPDYLDIPAFLR	u	-	-	3	Mix
FtsZ-FtsZ	380–51	**K**QAD	KTAVGQTIQIGSGITK	u	5	Intra	13	Mix
FtsZ	380–66	**K**QAD	TAVGQTIQIGSGIT**K**GLGAGANPEVGR	u	4	Intra	-	-
FtsZ-FtsZ	380–170	**K**QAD	LL**K**VLGR	u	3	Intra	5	Mix
FtsZ-FtsZ	380–367	**K**QAD	VVNDNAPQTA**K**EPDYLDIPAFLR	u	5	Intra	10	Intra
ZapA-FtsZ	42–51	LQDL**K**ER	**K**TAVGQTIQIGSGITK	15.4 ^a^	5	Inter		
ZapA-FtsZ	42–66	LQDL**K**ER	TAVGQTIQIGSGIT**K**GLGAGANPEVGR	16.8 ^a^	4	Inter		
ZapA-ZapA	42–42	LQDL**K**ER	LQDL**K**ER	26.3	2	Inter		
ZapA-ZapA	42–103	LQDL**K**ER	ITE**K**TNQNFE	23.9	3	Inter		
ZapA-ZapA	71–103	A**K**TR	ITE**K**TNQNFE	8.4	8	Inter		
ZapA-ZapA	71–69	A**K**TR	VTNEQLVFIAALNISYELAQE**K**AK	9.0	2	Inter		
ZapA-ZapA	103–103	ITE**K**TNQNFE	ITE**K**TNQNFE	22.3	1	Inter		

Intra, intraprotein cross-link; inter, interprotein cross-link; u, cross-link with the unstructured C-terminal tail. Linked residues are in bold font. ^a^ Distances are based on the HADDOCK model of ZapA and FtsZ as shown in Figure 7. ^b^ Spectral counts, number of times that the precursor was selected in data-dependent acquisition leading to identification of the cross-linked peptide.

**Table 2:** Morphology of TB28ΔzapA cells grown in Trypton Yeast (TY) at 37 °C with ZapA mutants expressed form plasmid and induced with 50 μM IPTG for six mass doublings.

ZapA Mutants	Cell Length ± S.E.M. (μm)	n ^a^	Cell Length >10 μm ± S.E.M. (%)	ZapA Fluorescence ^b^
TB28	4.13 ± 0.05	(2)4	0.1 ± 0.1	100
Empty vector	6.87 ± 0.87	(4)13	12.4 ± 4.3	38
Wild-type	5.02 ± 0.39	(5)15	2.3 ± 1.4	100
R13D	5.70 ± 0.70	(3)9	7.3 ± 3.2	95
D32A	4.81 ± 0.17	(2)3	1.0 ± 0.4	92
D32K	5.11 ± 0.04	(2)4	2.8 ± 1.5	106
N35A	4.54 ± 0.05	(2)3	0.6 ± 0.3	79
N35D	5.05 ± 0.09	(2)3	1.4 ± 1.0	68
Q39A	4.67 ± 1.23	(2)3	0.8 ± 0.3	82
Q39E	4.94 ± 0.12	(1)3	2.4 ± 0.6	104
Q39K	4.96 ± 0.08	(2)3	1.4 ± 0.8	88
K42A	5.22 ± 0.26	(2)5	3.2 ± 1.0	87
K42E	5.20 ± 0.35	(2)4	2.8 ± 1.7	98
R46E	5.85 ± 0.17	(2)5	5.8 ± 0.7	115
T48D	5.86 ± 0.37	(2)6	6.0 ± 1.9	67
T48R	4.60 ± 0.6	(2)5	2.3 ± 1.2	70
T50R, E51D	5.28 ± 0.16	(2)6	3.0 ± 0.7	75
E51K	6.74 ± 0.35	(3)7	10.9 ± 2.9	96
I56K	7.08 ± 0.41	(2)7	12.9 ± 2.3	99
E109K	5.58 ± 0.37	(3)7	4.7 ± 2.3	67

^a^ n is the number of individual repeats of the experiment and the number of technical repeats is given between brackets. The average length of the cells in the culture is based on the experimental and technical repeats. ^b^ The ZapA fluorescence is expressed as percentage of the fluorescence detected by the expression of the wild-type protein from plasmid. ZapA immunolabeling was performed two times and in some cases more than two times and the average ZapA concentration of these experiments is given. TB28 and TB28ΔzapA are MG12655 derivatives [10]. S.E.M., standard error of the mean.

## Supplementary Materials

Supplementary materials can be found at http://www.mdpi.com/1422-0067/19/10/2928/s1.
