# Supplementary material for: Mapping the Contact Sites of the Escherichia coli Division-Initiating Proteins FtsZ and ZapA by BAMG Cross-Linking and Site-Directed Mutagenesis
Source: Int J Mol Sci. 2018 Sep 26;19(10):2928. doi: 10.3390/ijms19102928 (PMC6213154; doi:10.3390/ijms19102928)
Supplement: Supplementary file 1 [file ijms-19-02928-s001.zip › ijms-354194-siTdB.docx]

**Mapping the contact sites of the *Escherichia coli* division initiating proteins FtsZ and ZapA by BAMG cross-linking and site directed mutagenesis**

**Supplementary Information**

This document concerns: Fig. S1 showing the polymerization behavior of the isolated FtsZ molecules and an immunoblot of the cross-linked products under the conditions that were used for the cross-linking experiments with BAMG. In addition, it is shown that the ^14^N and ^15^N-labeled FtsZ polymerizes equally well. Fig. S2 shows mass spectra of the identified cross-linked peptides. Fig. S3 gives an overview of the localization of FtsZ, ZapA mutants, and ZapB in a *zapA* chromosomal deletion strain. Fig. S4 shows that the ZapA mutants were not overproduced and a immunoblot showing the ZapA expression. Table S1 is an Excell sheet with annotated spectra of all identified cross-linked peptides. Table S2 contains the Haddock parameters for the model. Table S3 contains the interacting residues in the model of Figure 7.Table S4 lists the residues involved in contacts between ZapA and FtsZ in the best HADDOCK cluster (#5). Table S5 contains the used primers for the side directed mutagenesis of ZapA. Webfile S1 is the HADDOCK parameter web-file containing all input data and settings. PDB S1 contains the PDB coordinates of the best model (cluster 5.1).

**Supplementary Experimental Procedures**

*Bacterial strains and growth conditions*

FtsZ was isolated from *E. coli* strain BL21(DE3) pRRE6 [1]. DH5α [2] was used to isolate plasmid DNA. Strains TB28 and TB28Δ*zapA* [3] were used to determine whether ZapA mutants were able to complement the absence of wild type ZapA. Cells were grown in TY broth (10 g trypton, 5 g yeast extract, 5 g NaCl per liter, pH 7.0) or in glucose minimal medium (GB1) containing 6.33 g of K_2_HPO_4_.3H_2_O, 2.95 g of KH_2_PO_4_, 1.05 g of (NH_4_)_2_SO_4_, 0.10 g of MgSO_4_.7H_2_O, 0.28 mg of FeSO_4_.7H_2_O, 7.1 mg of Ca(NO_3_)_2_.4H_2_O, 4 mg of thiamine, 4 g of glucose and 50 mg of required amino acids, per liter, pH 7.0 at 28°C. For ^15^N-isotope labeling, Ca(NO_3_)_2_.4H_2_O was replaced by CaCl_2_ and (^15^NH_4_)_2_SO_4_ was used to grow the cells in GB1 medium for ^15^N-FtsZ isolation. Absorbance was measured at 450 nm (GB1) or 600 nm (TY) with a Biochrom Libra S70 spectrophotometer. Cells expressing ZapA were diluted 1:10000 in TY from an over-night culture and grown in a shaking water bath at 37°C to an OD_600_ of 0.3. Subsequently the cells were diluted to 0.005 in pre-warmed medium with IPTG as specified and continued to grow for 6 mass doublings.

*Microscopy and image analysis*

For immunolocalization imaging the cells were immobilized on 1% agarose [4], and photographed with a CoolSnap *fx* (Photometrics) CCD camera mounted on an Olympus BX-60 fluorescence microscope through an UPLANFl 100x/1.3 oil objective (Japan). Images were taken using modified acquisition software that used the program ImageJ by Wayne Rasband. In all experiments the cells were first photographed in the phase contrast mode, then with the Cy3 filter (U-MNG, ex. 530-550 nm). Next, the two photographs were hyperstacked, the phase contrast and fluorescence images were aligned, background was subtracted and the length and diameter of the cells were determined from the phase contrast images and the localization and intensity of the fluorescence signal was analyzed in the fluorescence images using the public domain program Coli-Inspector [5] written by Norbert Vischer (University of Amsterdam, https://sils.fnwi.uva.nl/bcb/objectj/), which is based on ImageJ by Wayne Rasband.

*Rationale for cross-linking experiments*

The binding of GTP at the interface of two FtsZ monomers initiates polymerization provided that a critical concentration of FtsZ is present [6-10]. GTP hydrolysis causes the depolymerization of the protofilaments. Consequently, to be able to cross-link ZapA to FtsZ protofilaments a minimal FtsZ concentration and GTP concentration is required. The purine of GTP contains a free amine group. Although this group is expected to have a lower reactivity towards N-hydroxysuccinimidyl esters than the ε-amino group of lysine, due to the presence of electron withdrawing N-atoms in the purine ring, we decided to determine with light scattering experiments the minimal GTP concentration that would still give sufficient FtsZ polymers and enough time to add the cross-linker. The optimal cross-linker concentration and cross-linking duration was determined using DSG (disuccinimidyl glutarate). DSG has the same spacer length and the same cross-linking reactivity as BAMG, the cross-linker finally used. Unlike BAMG, DSG is not cleaved under reducing conditions [11] making it more suitable for standard SDS-PAGE analysis.

**CROSS-LINKING**

*Isolation of cross-linked peptides*

A tryptic digest of a BAMG-cross-linked sample was brought to RT and the peptides were desalted on C18 reverse phase tips (TT3 TopTip, Glygen, capacity 1 mg), eluted with 50 μl 0.1% TFA in 50% ACN and added to 1.8 mg azido-reactive cyclooctyne (ARCO) coupled to polydimethyl acryamide (PL-DMA) beads in a 2 ml polypropylene Protein Lobind vial (Eppendorf). The sample was incubated 24 hrs at 40°C on a rotating device to covalently bind the cross-linked material to the ARCO beads.

The sample containing the ARCO beads was briefly centrifuged and washed subsequently for 10 min with 1 ml 0.1 M acetic acid in 50% ACN, 1 ml 0.6 M NaCl in 50% ACN, and 1 ml 0.1 M NH_4_HCO_3_, while rotating at RT. The peptides were eluted from the beads with 50 μl 5 mM tris(carboxyethyl)phosphine (TCEP) and 0.1 M NH_4_HCO_3_ in 50% ACN while rotating for 30 min at RT. The beads were centrifuged and the supernatant was collected in a 2 ml protein LoBindvial. The beads were washed for 10 min with 50 μl 0.1 M NH_4_HCO_3_ in 50% ACN and the supernatant was added to the 2 ml vial with the released material. Free thiols were alkylated with 20 mM iodoacetamide in 0.1 M NH_4_HCO_3_ by incubation in the dark for 30 min at RT. Subsequently, 0.7 ml 0.2% TFA was added to the sample to lower the pH to ~2.0 and the ACN concentration to less than 7%. Peptides were desalted with TT3 Toptips (Glygen) and after three washing steps with 100 μl 0.1% TFA eluted using 50 μl 0.1% TFA in 75% ACN. Peptides were dried in a vacuum centrifuge.

*Strong cation exchange (SCX) chromatography*

Fractionation by SCX chromatography of the material released from the ARCO beads was carried out as described before [12] with minor modifications using a Polysulfethyl Aspartamide^TM^ Column (2.1 mm ID, 10 cm length) (PolyLC Inc, Columbia, USA) operated on an Ultimate HPLC system (LC Packings, Amsterdam, The Netherlands). In brief, the peptides were dissolved in 50 μl 0.1% TFA in 75% ACN to which immediately 1 ml 10 mM potassium phosphate buffer (pH 2.72) in 25% ACN was added, followed by loading on the column. The flow rate was 0.1 ml.min^-1^. After washing for 40 min with buffer A (50 mM KCl in 10 mM potassium phosphate buffer (pH 2.9) in 25% ACN), the peptides were eluted in two steps; for 20 min with 10% buffer B (buffer A with 500 mM KCl), for 8 min with 50% buffer B, for the next 10 min with a salt gradient linearly increasing to 100% buffer B and finally for 10 min with 100% buffer B. The eluate was collected in a low salt fraction enriched in Type 0 and in Type 1 cross-links (elution at 10% buffer B) and a high salt fraction enriched in Type 2 cross-links (elution at 50% buffer B). The high and low salt fractions were diluted 4 times with 0.1% TFA and the peptides were desalted using C18-Omix 10-100 μl pipette tips (Agilent). Bound material was eluted using 50 μl 0.1% TFA in 75% ACN and each sample was divided over four 200 μl glass insert vials (Grace), dried in a vacuum centrifuge and stored at 4°C.

*Reversed phase liquid chromatography coupled to mass spectrometry (LC-MS/MS)*

MS/MS data were acquired as described before [12] using an ApexUltra Fourier transform ion cyclotron resonance mass spectrometer (Bruker Daltonic, Bremen, Germany) equipped with a 7 T magnet and an Apollo II dual source coupled to an Ultimate 3000 HPLC system with an Acclaim PepMap 100 C18 analytical column with 3-μm particle size, 100-Å pore size, 75-μm inner diameter and 250 mm length (Thermo Scientific, Etten-Leur, The Netherlands) operated at a flow rate of 300 nl/min. Dry samples in glass insert vials were reconstituted with a solution containing 0.1% TFA in 5% acetonitrile just prior to LC-MS/MS analysis to prevent possible premature aggregation of peptides [12]. Some samples were also analyzed by LC-MS/MS using a Q-TOF mass spectrophotometer [12].

*Identification of unmodified peptides and type 0 and type 1 cross-linked peptides*

The Uniprot *Escherichia coli* database (release date June 2012) was searched by Mascot [13] of unmodified peptides, type 0 cross-linked peptides and type 1 cross-linked peptides at 15 ppm mass accuracy for precursor ions and 0.015 Da mass accuracy for fragment ions. For unmodified peptides, up to one missed cleavage was included in the search parameters, while for type 0 and type 1 cross-links two, resp. three missed cleavages were allowed. Alkylation by iodoacaetamide at C was used as a fixed modification. Four variable modifications were included in the search parameters, i.e., (i) modification at K with the cross-linker remnant of which one of the activated esters has become hydrolyzed or (ii) has reacted with Tris, (iii), type 1 cross-linking at K and (iv), oxidation at M.

**FIG. S1 related to experimental procedures and Fig. 1.** Optimized conditions for the polymerization and cross-linking of FtsZ and ZapA. A. Light scattering experiments in a cuvette containing 3.4 µM FtsZ in 50 mM HEPES pH 7.5, 50 mM KCl and 5 mM MgCl_2_ at 30°C (solid and dotted lines) or the same components but additionally 10 mM CaCl_2_ (dashed line). After stabilization of the baseline for 50 sec buffer either only GTP (solid line) or ZapA and GTP (dotted line and dashed line) were added at with the arrows indicated time points. Final concentrations were 60 μM GTP under non-bundling conditions and 3.75 μM under bundling conditions (*i.e.* in the presence of CaCl_2_, dotted line) and 6.8 µM ZapA. B. Immunoblots developed with antibodies against ZapA (left panel) and FtsZ (right panel), respectively. Lanes: 1, Molecular mass marker; 2, pure FtsZ; 3, pure ZapA and 4, cross-linked FtsZ and ZapA under non-bundling conditions after 5 min incubation with 100 μM DSG. C. Light scattering experiment using a cuvette containing 60 μM GTP in 50 mM HEPES pH 7.5, 50 mM KCl and 5 mM MgCl_2_ at 30 °C. After about 100 s ^14^N-FtsZ or ^15^N-FtsZ were added to a final concentration of 3.4 µM. The traces show two experiments for each isotope.

**FIG. S2 related to Table 1.** Mass spectra of ^14^N- +^15^N-labelled cross-linked FtsZ peptides. cross-linked peptides were detected under two polymerizing conditions in the presence of GTP: (i) in the presence of ZapA and in the absence of Ca^2+^, indicated by + ZapA, and (ii) in the absence of ZapA and in the presence of Ca^2+^, indicated by - ZapA. ^14^N-labelled peptides are denoted light peptides (L); ^15^N-labeled peptides are denoted heavy peptides (H). Blue peaks, both peptides are light (LL); red peaks, both peptides are heavy (HH); green peaks, light peptide A cross-linked to heavy peptide B (LH); yellow peaks, heavy peptide A cross-linked to light peptide B (HL). Sequences of composing peptides are depicted in the order peptide A and peptide B. Relative intensities of the four combinations LL, LH, HL and HH are expressed as percentages of the combination of highest intensity in font colors corresponding to peak colors. The percentage of interprotein cross-links is defined as: (intensity of LH + intensity of HL)/(intensity of LL + intensity of HH)/100%.

**Fig. S3 related to Table 2 and Fig 5. A.** Titration IPTG concentration to induced expression from plasmid to complement the parental strain TB28. Wild type ZapA and indicated mutants were expressed form plasmid in strain TB28∆*zapA* to determine which IPTG concentration was able to recreate the morphology of the parental strain TB28. Since we wanted to avoid overexpression and interaction with FtsZ of ZapA mutants with a lower affinity, all experiment were performed with 50 µm IPTG. **B.** Immunoblot showing the amount of ZapA produced for the wild type ZapA expressed from plasmid, the empty vector that did not express zapA and two ZapA mutants. The bands above ZapA are non-specific interactions with the secondary antibody. For the immunolabeling different secondary antibodies where used that do not give background in the cells (see materials and methods).

| TB28  Dia-map | TB28  FL-map FtsZ | TB28  FL-map ZapA | TB28  FL-map ZapB |
| --- | --- | --- | --- |
|  |  |  |  |
| Brightness/contrast  FtsZ 3000  ZapA 4000  ZapB 190 |  |  |  |
| n = 2989  Cell length > 10µm: 0.1 ± 0.1 %, n=4 | | n = 1038 | n = 3528 |
|  |  |  |  |
| Wild type  Dia-map | Wild type  FL-map FtsZ | Wild type  FL-map ZapA | Wild type  FL-map ZapB |
|  |  |  |  |
| Brightness/contrast  FtsZ 100  ZapA 100  ZapB 120 |  |  |  |
| n=878  Cell length > 10µm: 2.3 ± 1.4% > n=15 | | n = 1364 | n = 809 |
|  |  |  |  |
| EV  Dia-map | EV  FL-map FtsZ | EV  FL-map ZapA | EV  FL-map ZapB |
|  |  |  |  |
| Brightness/contrast  FtsZ 100  ZapA 100  ZapB 120 |  |  |  |
| n=373  Cell length > 10µm: 12.4 % ± 4.3 %, n=13 | | n = 1162 | n = 556 |

| R13D  Dia-map | R13D  FL-map FtsZ | R13D  FL-map ZapA | R13D  FL-map ZapB |
| --- | --- | --- | --- |
|  |  |  |  |
| Brightness/contrast  FtsZ 3000  ZapA 4000  ZapB 3000 |  |  |  |
| n = 1565  Cell length > 10µm: 7.3 ± 3.2 %, n=9 | | n = 1347 | n = 1577 |
|  |  |  |  |
| D32K  Dia-map | D32K  FL-map FtsZ | D32K  type FL-map ZapA | D32K  FL-map ZapB |
|  |  |  |  |
| Brightness/contrast  FtsZ 3000  ZapA 4000  ZapB 3000 |  |  |  |
| n = 2034  Cell length > 10µm: 2.9 ± 1.3 %, n=4 | | n = 980 | n = 1582 |
|  |  |  |  |
| Q39E Dia-map | Q39E FL-map FtsZ | Q39E FL-map ZapA | Q39E FL-map ZapB |
|  |  |  |  |
| Brightness/contrast  FtsZ 3000  ZapA 4000  ZapB 190 |  |  |  |
| n = 1743  Cell length > 10µm: 2.4 ± 0.6 %, n=3 | | n = 996 | n = 2122 |
|  |  |  |  |
|  |  |  |  |
|  |  |  |  |
|  |  |  |  |
|  |  |  |  |
|  |  |  |  |
|  |  |  |  |
| K42A Dia-map | K42A FL-map FtsZ | K42A FL-map ZapA | K42A FL-map ZapB |
|  |  |  |  |
| Brightness/contrast  FtsZ 3000  ZapA 3000  ZapB 3000 |  |  |  |
| n = 1859  Cell length > 10µm: 3.2 ± 1.0 %, n = 5 | | n = 1877 | n = 1964 |
|  |  |  |  |
| K42E Dia-map | K42E FL-map FtsZ | K42E FL-map ZapA | K42E FL-map ZapB |
|  |  |  |  |
| Brightness/contrast  FtsZ 3500  ZapA 2000  ZapB 4500 |  |  |  |
| n =1688  Cell length > 10µm: 2.8 ± 1.7 %, n = 4 | | n = 2287 | n = 1124 |
|  |  |  |  |
| R46E Dia-map | R46E FL-map FtsZ | R46E FL-map ZapA | R46E  FL-map ZapB |
|  |  |  |  |
| Brightness/contrast  FtsZ 100  ZapA 100  ZapB 120 |  |  |  |
| n = 1347  Cell length > 10µm: 5.8 ± 0.7 %, n=5 | | n = 1289 | n= 1307 |
|  |  |  |  |
|  |  |  |  |
|  |  |  |  |
|  |  |  |  |
|  |  |  |  |
| T48D Dia-map | T48D FL-map FtsZ | T48D FL-map ZapA | T48D FL-map ZapB |
|  |  |  |  |
| Brightness/contrast  FtsZ 100  ZapA 100  ZapB 120 |  |  |  |
| n = 1842  Cell length > 10µm: 6.0 ± 1.9 %, n=6 | | n = 1339 | n = 1041 |
|  |  |  |  |
| T48R Dia-map | T48R FL-map FtsZ | T48R FL-map ZapA | T48R FL-map ZapB |
|  |  |  |  |
| Brightness/contrast  FtsZ 8000  ZapA 20000  ZapB 3000 |  |  |  |
| n = 2029  Cell length > 10µm: 2.3 ± 1.2 %, n =5 | | n = 845 | n =1402 |
|  |  |  |  |
| T50R E51D  Dia-map | T50R E51D  FL-map FtsZ | T50R E51D FL-map ZapA | T50R E51D  FL-map ZapB |
|  |  |  |  |
| Brightness/contrast  FtsZ 100  ZapA 100  ZapB 120 |  |  |  |
| n = 874  Cell length > 10µm: 3.0 ± 0.7 %, n = 6 | | n = 876 | n = 834 |
|  |  |  |  |
|  |  |  |  |
|  |  |  |  |
|  |  |  |  |
|  |  |  |  |
|  |  |  |  |
|  |  |  |  |
| E51K Dia-map | E51K FL-map FtsZ | E51K FL-map ZapA | E51K FL-map ZapB |
|  |  |  |  |
| Brightness/contrast  FtsZ 1000  ZapA 15000  ZapB 3000 |  |  |  |
| n = 724  Cell length > 10µm: 10.9 ± 2.9, n= 7 | | n = 946 | n =1176 |
|  |  |  |  |
| I56K Dia-map | I56K FL-map FtsZ | I56K FL-map ZapA | I56K FL-map ZapB |
|  |  |  |  |
| Brightness/contrast  FtsZ 1000  ZapA 15000  ZapB 3000 |  |  |  |
| n = 812  Cell length > 10µm: 12.9 ± 2.3, n=7 | | n = 1024 | n =1042 |
|  |  |  |  |
| E109K Dia-map | E109K FL-map FtsZ | E109K FL-map ZapA | E109K FL-map ZapB |
|  |  |  |  |
| Brightness/contrast  FtsZ 1000  ZapA 15000  ZapB 5000 |  |  |  |
| n = 709  Cell length > 10µm: 4.7 ± 2.3 %, n=7 | | n = 2187 | n =960 |

**Fig. S4 related to Table 2.** Map of diameter and map of fluorescence profiles and average fluorescence profile of FtsZ, ZapA and ZapB of TB28∆*zapA* expressing ZapA mutants from plasmid. The cells were grown exponentially in rich medium at 37°C and the OD_600_ was kept below 0.3. Expression of the mutants was induced with 50 µM IPTG for 6 mass doublings. Cells were fixed and immunolabeled as described in the experimental procedures. Of each sample, from left to right the map of diameter of the FtsZ labeled sample, the map of fluorescence of the FtsZ labeled sample, the map of fluorescence of the ZapA labeled sample, and the map of fluorescence of the ZapB labeled sample is shown. The cells are sorted according to their length in the maps (small cells on top and the longest cell on the bottom of the map). Number of analyzed cells are indicated and the percentage of cells with a cell length of more than 10 µm and the s.e.m. from a number (n) of experiments as indicated is shown. Brightness and contrast reflect the grey values within the map of fluorescence. EV is TB28∆zapA containing the plasmid that does not express any protein.

**TABLE S1 related to Table 1.** Excell sheet with annotated spectra of all identified cross-linked peptides. The *m/z* values of assigned fragments originating from peptide bond cleavages in peptide A (yellow) and in peptide B (orange) or cleavage of the amide bonds of the cross-link (blue) are highlighted. In the left two columns, the observed *m/z* values of fragment ions and corresponding signal intensities are depicted. The *m/z* values of fragment ions are calculated for primary fragments (upper central panel) and secondary fragments of unmodified peptides A and B (middle central panel) and peptides A and B modified by a remnant of the cross-linker after reaction with cycloctyne (lower central panel). Right panel, calculated *m/z* values of modified and unmodified peptides A and B after cleavage of a cross-link amide bond. Highlighted black numbers, assigned unambiguous fragments. cross-linked lysines are highlighted red. Blue numbers, ambiguous fragments.

**Table S2.** Definition of restraints for HADDOCK

| *Cross-link distance restraints* | *CA-CA distance range* |
| --- | --- |
| - ZapA LYS 42 or 242 – FtsZ LYS 51 or 451 or 851 - ZapA LYS 42 or 242 – FtsZ LYS 66 or 466 or 866 | 20-0Å  20-0Å |
| *Active residues on ZapA* *(residue numbering shifted by 200/400/600 for the 2^nd^, 3^rd^ and 4^th^ monomers, respectively)* | |
| - ARG 13 or 213 - ARG 46 or 246 - THR 48 or 248 - GLU 51 or 251 |  |
|  |  |
| *Passive residues (solvent accessible ones on the central monomer of the trimeric FtsZ repeat – residue numbering shifted by 400)* | |
| 401,402,403,405,406,407,409,410,411,412,420,421,424,431,432,433,435,436,437,438,451,453,454,455,456,462,463,464,466,473,475,479,482,483,485,486,487,489,490,493,494,496,503,518,521,522,523,524,536,537,540,541,544,548,551,554,555,557,570,571,572,573,574,575,576,577,580,582,583,586,587,591,603,604,605,607,612,614,615,618,619,621,627,631,632,633,634,637,638,641,642,645,647,651,652,653,655,656,658,668,669,671,672,673,676,684,685,687,688,689,691,700,701,703,704,707,717,720,721,723,724,727,728 | |

**Table S3.** HADDOCK clustering statistics. The various terms are calculated as the average over the top 4 members of each clusters. Only the top10 clusters (out of 17) is reported here.

| **Cluster#** | **HADDOCK score**  [a.u.] | **Size** | **RMSD from best model** [Å] | **E_vdw_^a^**  [kcal mol^-1^] | **E_elec_^a^**  [kcal mol^-1^] | **E_desol_^b^**  [kcal mol^-1^] | **Restraint energy^c^**  [kcal mol^-1^] | **BSA^d^**  [Å^2^] |
| --- | --- | --- | --- | --- | --- | --- | --- | --- |
| 5 | -169.2±11.2 | 12 | 1.0±0.6 | -71.6±7.0 | -231.4±21.6 | -52.7± 9.7 | 14.4±1.0 | 2260±106 |
| 4 | -159.9± 7.6 | 13 | 5.6±0.0 | -49.2±4.4 | -292.1± 4.4 | -53.3± 7.3 | 9.7±1.1 | 1832±107 |
| 17 | -156.2±16.7 | 4 | 6.0±0.0 | -51.1±6.2 | -271.1±61.7 | -52.2± 6.6 | 14.5±1.5 | 1821±108 |
| 7 | -151.5±12.6 | 10 | 7.6±0.1 | -74.2±4.1 | -163.4±34.0 | -46.1±10.2 | 14.8±2.3 | 2171±111 |
| 2 | -143.1± 2.4 | 18 | 23.6±0.3 | -32.6±4.4 | -237.4±16.1 | -63.9± 7.2 | 8.7±2.1 | 1066±104 |
| 16 | -139.4±16.6 | 4 | 21.5±0.5 | -57.2±8.2 | -230.4±23.7 | -37.0±10.3 | 8.7±2.3 | 1502± 84 |
| 10 | -138.9± 7.8 | 6 | 8.0±0.9 | -45.5±2.3 | -234.1±73.8 | -47.9± 8.9 | 13.4±1.8 | 1722±151 |
| 1 | -137.7± 4.3 | 36 | 7.5±0.0 | -36.2±3.0 | -187.0± 9.1 | -65.4± 7.6 | 13.3±3.3 | 1334± 34 |
| 8 | -136.2±12.1 | 9 | 9.4±0.3 | -36.7±3.4 | -192.2±17.5 | -63.3±11.1 | 22.4±5.0 | 1491±118 |
| 6 | -135.2± 2.2 | 11 | 7.0±0.0 | -51.5±7.5 | -183.7±28.8 | -47.7± 2.7 | 7.7± 1.1 | 1624± 83 |

a) Intermolecular van der Waals and electrostatic energies calculated with the OPLS force field [14] and an 8.5Å non-bonded cutoff
b) Empirical desolvation potential [15].
c) Distance restraint energy. The terms comprises both the cross-links and the mutations.
d) Buried surface area

**Table S4** related to figure 7. Residues involved in contacts between ZapA and FtsZ in the best HADDOCK cluster (#5). Contacts (calculated using LIGPLOT [16] are listed if present in at least 3 out of the top4 models of cluster 5.

| **Residue** | **ZapA chain** | **residue** | **FtsZ chain** |
| --- | --- | --- | --- |
| ARG 13 | A (lime) | GLU 493 | B orange |
| ARG 46 | A | GLU 406 | B |
| ASN 49 | A | GLY 494 | B |
| GLU 51 | A | LYS 414 | B |
| ARG 13 | B | GLU 438 | B |
| ARG 16 | B | THR 408 | B |
| ARG 16 | B | ASP 410 | B |
| LYS 42 | B | GLY 455 | B |
| THR 48 | B (green) | GLN 456 | B |
| ASN 49 | B | ALA 490 | B |
|  |  |  |  |

**TABLE S5** **related to Table 2** primers used in this study

| Name | Sequence in 5’-3’ notation (accompanying change in restriction site) |
| --- | --- |
| ZapAR13D F | GATATCCAAATTTTTGGCgaTTCcCTGaGgGTGAACTGCCCGCCTG (*Bsu*36I) |
| ZapAR13D R | CAGGCGGGCAGTTCACcCtCAGgGAAtcGCCAAAAATTTGGATATC |
| ZapAD32A F | GTTGAATCAGGCAGCGgccGATCTGAACCAACGtcTaCAAGATCTGAAAGAAC (*Acc*I) |
| ZapAD32A R | CTTCTTTCAGATCTTGtAgaCGTTGGTTCAGATCggcCGCTGCCTGATTCAAC |
| ZapAD32E F | GTTGAATCAGGCAGCGaagGATCTGAACCAACGtcTaCAAGATCTGAAAGAAC (*Acc*I) |
| ZapAD32E R | CTTCTTTCAGATCTTGtAgaCGTTGGTTCAGATCcttCGCTGCCTGATTCAAC |
| ZapAN35A F | CCAACGGTTGCAAGATC(PvuII) |
| ZapAN35A R | GATCTTGCAACCGTTGGgcCAGATCGTCaGCTGCCTGATTCAACG |
| ZapAN35D F | CTGAAaGAACGCACTAG (BglII lost) |
| ZapAN35D R | CTAGTGCGTTCtTTCTAG |
| ZapAQ39A F | GATCTGAAAGAACGCAC (*BglII lost)*) |
| ZapAQ39A R | GTGCGTTCTTTCAGATCgccAaCCGTTGGTTCAGATC |
| ZapAQ39E F | CGTTGAATCAGGCAGCtGACGATCTGAACCAACGGcTcgAgGATCTGAAAGAACGCAC (*Pvu*II) |
| ZapAQ39E R | GTGCGTTCTTTCAGATCcTcgAgCCGTTGGTTCAGATCGTCaGCTGCCTGATTCAACG |
| ZapAQ39K F | CTGAACCAACGGTTGaAgGATCTGAAAGAACGCACccGgGTCACAAATACTGAAC (*Sma*I) |
| ZapAQ39K R | GTTCAGTATTTGTGACcCggGTGCGTTCTTTCAGATCcTtCAACCGTTGGTTCAG |
| ZapAK42A F | CAACGGTTGCAAGATCTGgcgGAACGCACTcGAGTCACAAATACTGAAC (*Xho*I) |
| ZapAK42A R | GTTCAGTATTTGTGACTCgAGTGCGTTCcgcCAGATCTTGCAACCGTTG |
| ZapAK42E F | CAACGGTTGCAAGATCTGgAAGAACGCACTcGAGTCACAAATACTGAAC (*Xho*I) |
| ZapAK42E R | GTTCAGTATTTGTGACTCgAGTGCGTTCTTcCAGATCTTGCAACCGTTG |
| ZapAR46E F | CTGAAAGAACGCACTgaAGTCACAAATACTG |
| ZapAR46E R | CAGTATTTGTGACTtcAGTGCGTTCTTTCAG |
| ZapAT48D F | GAACGCACTAGAGTCgacAATACTGAACAG (*Sal*I) |
| ZapAT48D R | CTGTTCAGTATTgtcGACTCTAGTGCGTTC |
| ZapAT48R F | CGCACTAGAGTCA*g*AAATACTGAACAGTTG |
| ZapAT48R R | CAACTGTTCAGTATTT*c*TGACTCTAGTGCG |
| ZapAT50RE51D F | CGCACTAGAGTCACAAATcgTGAtCAGTTGGTCTTC (*Bcl*I) |
| ZapAT50RE51D R | GAAGACCAACTGaTCAcgATTTGTGACTCTAGTGCG |
| ZapAE51K F | ACTcGAGTCACAAATACTaAACAGTTGGTCT (*Xho*1) |
| ZapAE51K R | ACCAACTGTTtAGTATTTGTGACTCgAGT |
| ZapAI56K F | GAACAaTTGGTCTTCAagGCCGCATTGAAT (*Mfe*I) |
| ZapAI56K R | TCAATGCGGCctTGAAGACCAAtTGTTC |
| ZapAE109K F | AAAACTTTcgaTGATGGTCcACgTGCAGGCA (*Pml*1) |
| ZapAE109K R | TGCCTGCAcGTgGACCATCAacgAAAGTTTTGGTT |

Codon changes to introduce restriction enzyme sites without changing the amino acid sequence and to introduce the indicated mutation (s) are shown in red. Restriction sites are underlined.

**SUPPLEMENTARY REFERENCES**

1. Läppchen, T.; Hartog, A. F.; Pinas, V. A.; Koomen, G.-J.; Blaauwen, den, T. GTP analogue inhibits polymerization and GTPase activity of the bacterial protein FtsZ without affecting its eukaryotic homologue tubulin. *Biochemistry* **2005**, *44*, 7879–7884.

2. Hanahan, D. Studies on transformation of *Escherichia coli* with plasmids. *J. Mol. Biol.* **1983**, *166*, 557–580.

3. Galli, E.; Gerdes, K. Spatial resolution of two bacterial cell division proteins: ZapA recruits ZapB to the inner face of the Z-ring. *Mol Microbiol* **2010**, *76*, 1514–1526.

4. Koppelman, C.-M.; Aarsman, M. E. G.; Postmus, J.; Pas, E.; Muijsers, A. O.; Scheffers, D.-J.; Nanninga, N.; Blaauwen, den, T. R174 of *Escherichia coli* FtsZ is involved in membrane interaction and protofilament bundling, and is essential for cell division. *Mol Microbiol* **2004**, *51*, 645–657.

5. Vischer, N. O. E.; Verheul, J.; Postma, M.; van den Berg van Saparoea, B.; Galli, E.; Natale, P.; Gerdes, K.; Luirink, J.; Vollmer, W.; Vicente, M.; Blaauwen, den, T. Cell age dependent concentration of Escherichia coli divisome proteins analyzed with ImageJ and ObjectJ. *Front Microbiol* **2015**, *6*, 586.

6. Sossong, T. M.; Brigham-Burke, M. R.; Hensley, P.; Pearce, K. H. Self-Activation of Guanosine Triphosphatase Activity by Oligomerization of the Bacterial Cell Division Protein FtsZ. *Biochemistry* **1999**, *38*, 14843–14850.

7. González, J. M.; Vélez, M.; Jiménez, M.; Alfonso, C.; Schuck, P.; Mingorance, J.; Vicente, M.; Minton, A. P.; Rivas, G. Cooperative behavior of Escherichia coli cell-division protein FtsZ assembly involves the preferential cyclization of long single-stranded fibrils. *Proc Natl Acad Sci USA* **2005**, *102*, 1895–1900.

8. Caplan, M. R.; Erickson, H. P. Apparent cooperative assembly of the bacterial cell division protein FtsZ demonstrated by isothermal titration calorimetry. *J Biol Chem* **2003**, *278*, 13784–13788.

9. Chen, Y.; Bjornson, K.; Redick, S. D.; Erickson, H. P. A rapid fluorescence assay for FtsZ assembly indicates cooperative assembly with a dimer nucleus. *Biophys J* **2004**, *88*, 505–514.

10. Romberg, L.; Levin, P. A. Assembly dynamics of the bacterial cell division protein FtsZ: poised at the edge of stability. *Annu Rev Microbiol* **2003**, *57*, 125–154.

11. Kasper, P. T.; Back, J. W.; Vitale, M.; Hartog, A. F.; Roseboom, W.; de Koning, L. J.; van Maarseveen, J. H.; Muijsers, A. O.; de Koster, C. G.; de Jong, L. An aptly positioned azido group in the spacer of a protein cross-linker for facile mapping of lysines in close proximity. *ChemBioChem* **2007**, *8*, 1281–1292.

12. Buncherd, H.; Nessen, M. A.; Nouse, N.; Stelder, S. K.; Roseboom, W.; Dekker, H. L.; Arents, J. C.; Smeenk, L. E.; Wanner, M. J.; van Maarseveen, J. H.; Yang, X.; Lewis, P. J.; de Koning, L. J.; de Koster, C. G.; de Jong, L. Selective enrichment and identification of cross-linked peptides to study 3-D structures of protein complexes by mass spectrometry. *Journal of Proteomics* **2012**, *75*, 2205–2215.

13. Perkins, D. N.; Pappin, D. J.; Creasy, D. M.; Cottrell, J. S. Probability-based protein identification by searching sequence databases using mass spectrometry data. *Electrophoresis* **1999**, *20*, 3551–3567.

14. Jorgensen, W. L.; Tirado-Rives, J. The OPLS [optimized potentials for liquid simulations] potential functions for proteins, energy minimizations for crystals of cyclic peptides and crambin. *J Am Chem Soc* **1988**, *110*, 1657–1666.

15. Fernández-Recio, J.; Totrov, M.; Abagyan, R. Identification of Protein–Protein Interaction Sites from Docking Energy Landscapes. *J Mol Biol* **2004**, *335*, 843–865.

16. Wallace, A. C.; Laskowski, R. A.; Thornton, J. M. LIGPLOT: a program to generate schematic diagrams of protein-ligand interactions. *Protein Eng* **1995**, *8*, 127–134.
